# What measured blood loss tells us about postpartum bleeding: a systematic review

**DOI:** 10.1111/j.1471-0528.2010.02567.x

**Published:** 2010-06

**Authors:** NL Sloan, J Durocher, T Aldrich, J Blum, B Winikoff

**Affiliations:** Gynuity Health ProjectsNew York, NY, USA

**Keywords:** Postpartum blood loss, postpartum haemorrhage, third stage of labour

## Abstract

**Background:**

Meta-analyses of postpartum blood loss and the effect of uterotonics are biased by visually estimated blood loss.

**Objectives:**

To conduct a systematic review of measured postpartum blood loss with and without prophylactic uterotonics for prevention of postpartum haemorrhage (PPH).

**Search strategy:**

We searched Medline and PubMed terms (labour stage, third) AND (ergonovine, ergonovine tartrate, methylergonovine, oxytocin, oxytocics or misoprostol) AND (postpartum haemorrhage or haemorrhage) and Cochrane reviews without any language restriction.

**Selection criteria:**

Refereed publications in the period 1988–2007 reporting mean postpartum blood loss, PPH (≥500 ml) or severe PPH (≥1000 ml) following vaginal births.

**Data collection and analysis:**

Raw data were abstracted into Excel by one author and then reviewed by a co-author. Data were transferred to SPSS 17.0, and copied into RevMan 5.0 to perform random effects meta-analysis.

**Main results:**

The distribution of average blood loss (29 studies) is similar with any prophylactic uterotonic, and is lower than without prophylaxis. Compared with no uterotonic, oxytocin and misoprostol have lower PPH (OR 0.43, 95% CI 0.23–0.81; OR 0.73, 95% CI 0.50–1.08, respectively) and severe PPH rates (OR 0.61, 95% CI 0.29–1.29; OR 0.74, 95% CI 0.52–1.04, respectively). Oxytocin has lower PPH (OR 0.65, 95% CI 0.60–0.70) and severe PPH (OR 0.71, 95% CI 0.56–0.91) rates than misoprostol, but not in developing countries.

**Conclusion:**

Oxytocin is superior to misoprostol in hospitals. Misoprostol substantially lowers PPH and severe PPH. A sound assessment of the relative merits of the two drugs is needed in rural areas of developing countries, where most PPH deaths occur.

## Introduction

Haemorrhage is the single leading cause of maternal mortality.^1^ Postpartum haemorrhage (PPH) is most often attributed to uterine atony.^2^ Most births and maternal deaths occur in Africa and Asia, where home deliveries are common, infrastructure and transportation are limited, and where birth attendants are scarce or inadequately prepared to prevent and treat PPH.^3^ In such settings haemorrhage accounts for ≥30% of maternal deaths.^1^ The United Nation’s Millennium Development goal 5, to reduce 75% of maternal mortality by 2015, cannot be reached without the successful management of PPH.^4^,^5^

The conventional definition of PPH is a blood loss of ≥500 ml in the first 24 hours after delivery.^6^,^7^ By stimulating uterine muscle tone, prophylactic uterotonics reduce the incidence of PPH.^2^,^8^,^9^ Several factors influence PPH rates, including whether blood loss is measured, how the third stage of labour is managed (e.g. the provision of uterotonic, uterine massage and controlled cord traction), obstetric interventions carried out (e.g. episiotomy and mode of delivery), and study population (sample size, parity, urban/rural or facility/home delivery, and level of facility).^10^ Most clinicians (and studies) classify obstetric blood loss by visual estimation. Visually (clinically) assessed bleeding underestimates measured blood loss by an average of 100–150 ml, and substantially underestimates blood loss of ≥500 ml (by 30–50%).^11^–^16^

Underestimating blood loss ‘lowers’ PPH rates and the estimates of prevented PPH, as there is artificially less PPH to prevent. A recent systematic review found the prevalence of PPH was 10.55% in 19 studies that measured postpartum blood loss, compared with 7.23% in 22 studies where blood loss was estimated visually, suggesting a large underestimation of PPH.^10^ Thus, in meta-analyses such as the Cochrane reviews of the efficacy of prophylactic uterotonics to reduce postpartum blood loss and prevent its sequellae, the proportion of studies and subjects where blood loss was visually rather than objectively measured influences the PPH and severe PPH rates, and thus influences the estimates (relative risks or odds ratios) of the effectiveness of uterotonic agents in preventing or treating obstetric haemorrhage.^2^,^8^,^17^–^19^

Most women experiencing a loss of ≥500 ml of blood (PPH) do not receive clinical intervention or experience serious consequences.^10^,^20^,^21^ In fact, some suggest that the 500-ml definition of PPH should be considered an alert level, and that PPH may be better defined as the volume of blood loss requiring intervention to avert serious sequellae.^22^,^23^ Accordingly, a re-evaluation of PPH guidelines has been recommended.^24^–^26^ This article presents information about average blood loss, and the incidence of PPH (≥500 ml) and severe PPH (≥1000 ml) in studies where blood loss was measured, to clarify what we know about postpartum blood loss among women who received and did not receive uterotonic prophylaxis during the third stage of labour.

## Methods

### Searching

**Figure 1:**
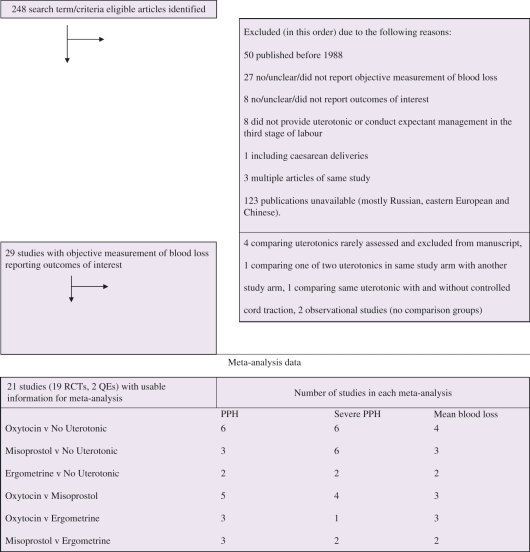
Studies reviewed and included in the meta-analyses.

Nearly 250 observational and experimental studies published up to 31 December 2007 were identified by Medline and PubMed online search engines using the following search terms: (labour stage, third) AND (ergonovine, ergonovine tartrate, methylergonovine, oxytocin, oxytocics or misoprostol) AND (postpartum haemorrhage or haemorrhage), without language restriction (Figure 1). Articles were also identified and reviewed if cited by the Cochrane reviews on management of the third stage of labour.^2^,^8^,^17^,^18^

### Inclusion and exclusion criteria

Studies were retained if there was objective measurement of blood loss after delivery, regardless of the duration of the blood measurement, augmentation or induction in the first or second stages of labour, or if other components of active management of the third stage of labour (AMTSL) were implemented (Figure 1). Articles published before 1 January 1988, with uncertain blood measurement, including one retrospective article,^27^ or articles published in journals that could not be accessed were excluded. Studies including caesarean deliveries were excluded to avoid biased comparisons should blood loss vary by delivery mode.^10^ However, studies with twin deliveries were included as twin and higher order births are relatively rare events. This review includes all eligible studies regardless of sample size. Twenty-three of the 59 study arms (39%) had sample arms of ≤200.

### Assessment of methodological quality

Each study was classified as a randomised controlled trial (RCT), quasi-experiment (QE) or observational (Obs). Study group allocation concealment was classified as: adequate (i) if a method such as consecutively numbered sealed opaque envelopes was used; unclear (ii) if the concealment technique was not described; or inadequate (iii) if there was an open list of random numbers or no random assignment (e.g. QE) was used.

### Data abstraction

All relevant raw data were abstracted from each eligible study by a single reviewer, and then reviewed by a co-author. Disagreements were resolved through verification against the publication and discussion. Data were then transferred to SPSS 17.0 and copied from the data extraction form into Review Manager (RevMan) 5.0 data analysis tables (The Cochrane Collaboration, 2008; The Nordic Cochrane Centre, Copenhagen, Denmark).

### Study characteristics

Twenty-nine articles were eligible for the review.^24^–^26^,^28^–^53^Table 1 presents their characteristics (location, sample size, blood measurement technique and components of AMTSL implemented). Studies were conducted in low-, middle- and high-income settings in Africa (*n* = 6), the Middle East (*n* = 4), Asia (*n* = 4) and Europe (*n* = 5), with one multi-country study conducted in all of these regions and Latin America. All but five of the studies were conducted in tertiary hospitals. The remaining five studies included home births in rural Gambia,^53^ home or village subcentre births in India,^24^ home and district hospital births in Vietnam,^32^ rural primary health centre births in Guinea-Bissau,^42^ and rural health centre births in India.^52^ Most studies measured blood loss by placing a bedpan underneath the parturient woman immediately after delivery, usually after the cord was clamped and cut. The collected blood was generally poured into a jar for volume measurement, and all soaked gauze pads were counted and weighed. Relatively few (*n* = 6) studies used the fairly new blood collection sheet or delivery drape, sometimes tied around the woman’s waist, with a funnel portion hanging between her legs, including the BRASSS-V Drape™ (a calibrated plastic sheet, Excellent Fixable Drapes, Madurai, Tamil Nadu, India).^54^ Two studies used the bedpan and linens method for some women and the drape for others. Most studies measured blood loss until active bleeding stopped, regardless of a pre-specified duration for blood measurement.

**Table 1:** Characteristics of studies reviewed (*n* = 29)

Study	Allocation concealment	Blood measurement	Sample *n*	Prophylactic regimen	CCT UM Dele Col	Notes	In meta analysis
Angola: Strand, 2005^26^	QE, NotBlind, C	PanLinen, 2 hours	782	Expectant		Uncomp, AugL	X
			814	Oxytocin, 10 iu IM	CCT		
Egypt: Abdel-Aleem, 2006^48^	RCT, NotBlind, RanEnv, B	Drape, 1 hour	102	Oxytocin, 10 iu IM or IV	CCT		
			98	Oxytocin, 10 iu IM or IV	UM		
Egypt: Prata, 2006^34^	Obs, NotBlind, C	Drape, 4 hours	1180	Misoprostol, 600 μg, O	CCT, UM		
France: Benchimol, 2001^47^	RCT, NotBlind, RanEnv, B	Drape, 1 hour	220	Expectant		AugL	X
			196	Oxytocin, 2.5 iu bolus IV			
			186	Misoprostol, 600 μg, O			
Gambia, rural: Walraven, 2005^53^	RCT, Ran (block), Double, A	PanLinen, 1 hour	630	Misoprostol, 600 μg, O	CCT; UM for PPH tx		X
			599	Ergometrine, 4 mg, O			
Guinea Bissau, rural: Høj, 2005^42^	RCT, Ran, Double, A	Both, 1 hour	331	Expectant, placebo	CCT		X
			330	Misoprostol, 600 μg, SL	CCT		
Hong Kong: Lam, 2004^39^	RCT (versus Other), Ran, NotBlind, C	PanLinen, 0th hour	30	Misoprostol, 600 μg, SL	CCT	LR	
Hong Kong: Yuen, 1995^30^	RCT, Ran (versus Other ), Double, A	PanLinen, 0th hour	495	Oxytocin, 10 iu IM		AugL	
India, rural: Derman, 2006^24^	RCT, Ran (block), Double, A	Drape, 2 hours	808	Expectant, placebo			X
			812	Misoprostol, 600 μg, O			
India: Gupta, 2006^44^	RCT, Ran, Double, A	Drape, 1 hour	100	Oxytocin, 10 iu IM	CCT I, UM I		X
			100	Misoprostol, 600 μg, R	CCT I, UM I		
India: Verma, 2006^31^	RCT, Runsp, Double, B	Drape, 0th hour	100	Ergometrine, 200 μg IM			X
			100	Misoprostol, 400 μg SL			
India, rural: Vimala, 2004^52^	RCT, Ran, NotBlind, B	PanLinen, 0th hour	60	Ergometrine (nonHBP), 200 μg IV Oxytocin (HBP), 10 iu IV	CCT	LR	
			60	Misoprostol, 400 μg SL	CCT		
India: Zachariah, 2006^29^	RCT, Ran, NotBlind, B	Both, 0th hour	676	Ergometrine, 2 mg IV			X
			617	Oxytocin, 10 iu IM			
			730	Misoprostol, 400 μg, O			
Ireland: Begley, 1990^49^	RCT, RanEnvBlock, NotBlind, C	PanLinen, 0th hour	724	Expectant	CCT	LR, AugL	X
			705	Ergometrine, 0.5 mg IV	CCT		
Israel: Soriano, 1996^33^	Obs, NotBlind, C	PanLinen, 0th hour	524	Oxytocin, 10 iu IV	CCT	AugL	
Japan: Fujimoto, 2006^51^	QE, NotBlind, C	PanLinen, 2 hours	82	Oxytocin, 5 iu IV	CCT	LR	X
			95	Oxytocin, 5 iu IV	CCT		
			70	Ergometrine, 0.2 mg IV	CCT		
			79	Ergometrine, 0.2 mg IV	CCT		
Mozambique: Bugalho, 2001^46^	RCT, RUnsp, Double, B	PanLinen, 0th hour	329	Oxytocin, 10 iu IM			X
			323	Misoprostol, 400 μg, R			
The Netherlands, multicentre: De Groot, 1996^50^	RCT, Ran Double (versus Ergometrine), NotBlind (versus Oxytocin), B	PanLinen, 1 hour	143	Expectant, placebo (O)		LR	X
			146	Ergometrine, 0.4 μg, O			
			78	Oxytocin, 0.5 μg, IM			
The Netherlands: Poeschmann, 1991^35^	RCT, Runsp (block), Double, B	PanLinen, 1 hour	24	Expectant, placebo		Uncomp, LR	X
			28	Oxytocin, 5 iu IM			
South Africa: Bamigboye, 1998^28^	RCT, Ran, Single, A	PanLinen, 1 hour	272	Expectant, placebo		LR	X
			271	Misoprostol, 400 μg R			
South Africa: Hofmeyr, 1998^26^	RCT, Ran (block), Double, A	PanLinen, 1 hour	250	Expectant, placebo	CCT		X
			250	Misoprostol, 400 μg, O	CCT		
South Africa: Hofmeyr, 2001^43^	RCT, Ran (block), Double, A	PanLinen, 1 hour	300	Expectant, placebo	CCT		X
			300	Misoprostol, 600 μg, O	CCT		
Sweden: Nordstrom, 1997^25^	RCT, Ran, Double, A	PanLinen, 0th hour	487	Expectant, placebo		AugL	X
			513	Oxytocin, 10 iu IV			
Turkey: Ozkaya, 2005^36^	RCT, Ran, Double (versus R) Single (versus V), A	PanLinen, 1 hour	44	Expectant, placebo, R			X^g^
			45	Misoprostol, 400 μg, V			
			48	Misoprostol, 400 μg, R			
UAE: Khan, 1997^41^	RCT, Runsp, NotBlind, C	PanLinen, 0th hour	821	Oxytocin, DoseUnsp, IV			
			827	Oxytocin, 10 iu IM	CCT		
UK: Mitchell, 1993^38^	RCT (versus Other), Runsp, Double, B	PanLinen, 1 hour	230	Oxytocin, 5 iu IM	CCT		
Vietnam, rural: Tsu, 2006^32^	QE, NotBlind, C	PanLinen, 0th hour	2371	Expectant	CCT, UM	AugL	X
			1236	Oxytocin, 10 iu IM	CCT, UM		
Zimbabwe: Kundodyiwa, 2001^40^	RCT, Ran, Double, A	PanLinen, 0th hour	256	Oxytocin, 10 iu IM			X
			243	Misoprostol, 400 μg, R			
Multicentre Gulmezoglu, 2001^45^	RCT, Ran (centrally), Double, A	PanLinen, 1 hour	9230	Oxytocin, 10 iu IV or IM	CCT, UM	AugL	X
			9225	Misoprostol, 600 μg, O	CCT, UM		

The format used for study identifiers was as follows: country, author, year.

Concealment allocation code: randomised controlled trial (RCT); quasi-experiment (QE); double blinded (Double); single blinded (Single); not blinded (NotBlind); blinding method unspecified (BlindUnsp); compared with regimen (such as syntometrine) not included in manuscript analyses (versus Other); randomisation generated by computer or table (Ran); randomisation by drawn envelope containing treatments (RanEnv); randomisation method unspecified (Runsp); A, adequate; B, unclear; C, inadequate.

Dose in micrograms (μg); dose in international units (iu); placebo (P); unidentical placebo (PlaceboUn); dose unspecified (DoseUnsp); route of administration (B, buccal; IM, intramuscular; IV, intravenous; Oral, oral; Rec, rectal; SubL, sublingual; V, vaginal; tx, treatment).

Third-stage management technique (TSL technique): CCT, controlled cord traction; UM, uterine massage; I, indeterminate/’gave AMTSL’; Expectant, expectant management.

Blood loss measurement technique: PanLinen, bedpan/linens; Drape; Both, both bedpan/linens and drape; Pads, NumberHrs, number of hours of measured blood loss; 0th hour, other hours measured.

Notes: study included augmented or induced labour (AugL), low-risk sample (LR) and uncomplicated deliveries (Uncomp).

Misoprostol, 400 μg, V group not included in analysis to limit to double blind comparison.

### Analysis

The range of average postpartum blood loss, rates of PPH and severe PPH, and ratio of severe PPH to PPH is presented for all eligible studies. In controlled studies comparing different prophylactic regimens, the effects of the regimen used to manage the third stage of labour on PPH, severe PPH and average postpartum blood loss were analysed by random-effects meta-analysis to avoid assumptions about similarity of study design or interventions. This systematic review presents Mantel–Haenszel odds ratios (ORs) for dichotomous (PPH and severe PPH) outcome, mean differences in blood loss and 95% confidence intervals (CIs). Heterogeneity across trials is evaluated using the chi-square test as calculated in MetaView. Subgroup analyses are presented for methodologically adequate studies, and figures with subgroup summary statistics are presented to demonstrate effects in individual studies and their settings. Observational studies or studies that compare different mechanisms of providing a single uterotonic are not included in the meta-analysis. In one study, only the comparison of the double-blind route was included when multiple routes of administration were studied to avoid over-counting the comparison group. Data on methergine were excluded as methergine was rarely assessed. Analyses were not stratified by dose or route (intravenous, intramuscular injection, oral, vaginal or rectal) to avoid reducing the analyses to single studies.

### Role of the funding source

The funding source had no role in the study design, data collection, analysis, interpretation or report composition.

## Results

### Distribution of mean blood loss

**Figure 2:**
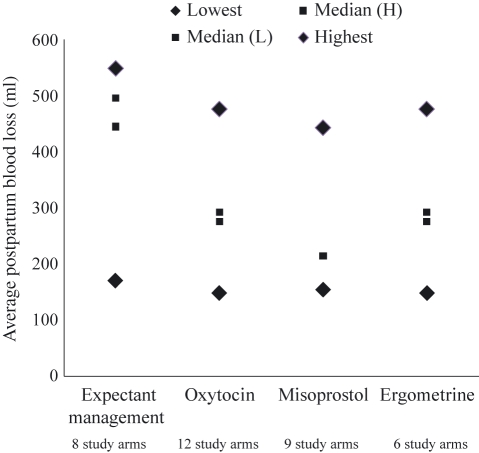
Median and range of average measured blood loss by regimen used to manage the third stage of labour.

The average blood loss ranged from 149 to 548 ml (Table 2; 16 studies). The highest average blood loss (range 171–548 ml) was among women managed without uterotonic prophylaxis (eight studies). The range of average blood loss was similar in women receiving any prophylactic uterotonic: 151–499 ml for oxytocin (ten studies, 12 study arms), 155–443 ml for misoprostol (eight studies, nine study arms), and 149–476 ml in women receiving ergometrine (five studies, six study arms). The 95% CI of each study arm was equivalent to 4–28% of the average blood loss of the study arm. The median average blood loss in women managed without a uterotonic is about 150–200 ml higher than for those provided with uterotonics, whereas the median and range of those managed with uterotonics are fairly uniform (Figure 2).

**Table 2:** Mean and 95% CI of measured postpartum blood loss by third-stage prophylactic regimen

Regimen	Study (author, year)	Mean blood loss (ml)	95% CI
No uterotonic	Angola: Strand, 2005^26^	445	424–476
	Guinea Bissau, rural: Høj, 2005^42^	496	475–517
	India, rural: Derman, 2006^24^	262	248–276
	Ireland: Begley, 1990^49^	235	218–251
	The Netherlands, multicentre: De Groot, 1996^50^	520	451–589
	The Netherlands: Poeschmann, 1991^35^	548	398–698
	Sweden: Nordstrom, 1997^25^	527	490–564
	Turkey: Ozkaya, 2005^36^	171	139–204
Oxytocin	Angola: Strand, 2005^26^	224	211–238
	Egypt: Abdel-Aleem, 2006^48^	282	248–315
	Egypt: Abdel-Aleem, 2006^48^	204	180–228
	India: Gupta, 2006^44^	151	137–165
	India: Zachariah, 2006^29^	183	173–193
	Japan: Fujimoto, 2006^51^	207	167–247
	Japan: Fujimoto, 2006^51^	288	244–332
	Mozambique: Bugalho, 2001^46^	157	142–172
	The Netherlands, multicentre: De Groot, 1996^50^	499	398–600
	The Netherlands: Poeschmann, 1991^35^	374	271–477
	Sweden: Nordstrom, 1997^26^	409	379–439
	UK: Mitchell, 1993^41^	252	229–275
Misoprostol	Gambia, rural: Walraven, 2005^53^	281	267–295
	Guinea Bissau, rural: Høj, 2005^42^	443	415–471
	India, rural: Derman, 2006^24^	214	204–224
	India: Gupta, 2006^44^	168	153–183
	India, rural: Vimala, 2004^31^	185	171–199
	India: Zachariah, 2006^29^	193	183–202
	Mozambique: Bugalho, 2001^46^	155	142–168
	Turkey: Ozkaya, 2005^36^	206	168–245
	Turkey: Ozkaya, 2005^36^	171	141–201
Ergometrine	Gambia, rural: Walraven, 2005^53^	292	278–306
	India: Zachariah, 2006^29^	188	178–198
	Ireland: Begley, 1990^49^	149	140–158
	Japan: Fujimoto, 2006^51^	338	289–387
	Japan: Fujimoto, 2006^51^	276	243–309
	The Netherlands, multicentre: De Groot, 1996^50^	476	421–531

### Distribution of PPH and severe PPH

The average PPH rate in the nine studies where women were managed expectantly (without uterotonic prophlyxis) ranged from 4 to 51% (Table 3). Where uterotonics were given, PPH ranged from 0 to 32% (17 studies, 19 study arms) for oxytocin, from 1 to 45% (12 studies) for misoprostol, and from 0 to 37% (seven studies, eight study arms) for ergometrine. Severe PPH ranged from 0.5 to 17% (12 studies) in women who were managed without prophylactic uterotonics, from 0.4 to 9% (12 studies, 13 study arms) for women managed with oxytocin, from 0 to 8% (11 studies) for women managed with misoprostol, and from 0 to 8% (five studies) for women managed with ergometrine.

**Table 3:** PPH (≥500 ml) and severe PPH (≥1000 ml) by third-stage prophylactic regimen

Prophylactic regimen	Study	% PPH	% Severe PPH	Ratio % Severe PPH to PPH
No uterotonic	Angola: Strand, 2005^26^	40.41	7.42	18.36
	France: Benchimol, 2001^47^	27.27	5.91	21.67
	Guinea Bissau, rural: Høj, 2005^42^	51.36	16.92	32.94
	India, rural: Derman, 2006^24^	12	1.24	10.33
	Ireland: Begley, 1990^49^	8.29	1.52	18.34
	The Netherlands, multicenter: de Groot, 1996^50^	38.46	11.19	29.10
	The Netherlands: Poeschmann, 1991^35^	41.67	12.5	30.00
	South Africa: Bamigboye, 1998^28^	NR	6.99	
	South Africa: Hofmeyr, 1998^23^	NR	9.2	
	South Africa: Hofmeyr, 2001^43^	NR	9.7	
	Sweden: Nordstrom, 1997^25^	35.93	8.83	24.58
	Vietnam: Tsu, 2006^32^	3.84	0.51	13.28
Oxytocin	Angola: Strand, 2005^26^	8.23	0.98	11.91
	Egypt: Abdel-Aleem, 2006^48^	4.08	NR	
	Egypt: Abdel-Aleem, 2006^48^	7.84	NR	41.38
	France: Benchimol, 2001^47^	14.79	6.12	41.38
	Hong Kong: Yuen, 1995^30^	12.12	2.02	16.67
	India: Gupta, 2006^44^	0	NR	
	India: Zachariah, 2006^29^	2.11	0.65	30.81
	Israel: Soriano, 1996^33^	9.73	NR	
	Japan: Fujimoto, 2006^51^	11.58	NR	
	Japan: Fujimoto, 2006^51^	7.32	NR	
	Multicentre Gulmezoglu, 2001^40^	13.53	2.85	21.06
	The Netherlands, multicentre: de Groot, 1996^50^	32.05	8.97	27.99
	The Netherlands: Poeschmann, 1991^35^	25.0	7.14	28.56
	Sweden: Nordstrom, 1997^25^	20.27	6.24	30.78
	UAE: Khan, 1997^41^	10.96	3.17	28.92
	UAE: Khan, 1997^41^	5.8	0.73	12.59
	UK: Mitchell, 1993^41^	7.39	0.43	5.82
	Vietnam: Tsu, 2006^32^	2.67	0.73	27.34
	Zimbabwe: Kundodyiwa, 2001^40^	13.28	1.95	14.68
Misoprostol	Egypt: Prata, 2006^34^	1.61	0.08	4.97
	France: Benchimol, 2001^47^	27.95	8.6	30.77
	Gambia, rural: Walraven, 2005^53^	10.97	0.32	2.92
	Guinea Bissau, rural: Høj, 2005^42^	45.45	11.21	24.66
	Hong Kong: Lam, 2004^39^	13.33	NR	
	India, rural: Derman, 2006^24^	6.4	0.25	3.91
	India: Gupta, 2006^44^	1		
	India: Verma, 2006^31^	1	NR	
	India, rural: Vimala, 2004^52^	3.33	0	0
	India: Zachariah, 2006^29^	2.6	0.14	5.38
	South Africa: Bamigboye, 1998^28^		4.81	
	South Africa: Hofmeyr, 1998^23^	NR	6.0	
	South Africa: Hofmeyr, 2001^43^	NR	9.0	
	Zimbabwe: Kundodyiwa, 2001^40^	15.23	3.7	24.29
	Multicentre Gulmezoglu, 2001^40^	19.46	3.97	20.40
Ergometrine	Gambia, rural: Walraven, 2005^53^	12.02	0.67	5.57
	India: Verma, 2006^31^	0	NR	
	India, rural: Vimala, 2004^52^	0	0	0
	India: Zachariah, 2006^29^	2.96	0.89	30.07
	Ireland: Begley, 1990^49^	1.99	0.14	7.04
	Japan: Fujimoto, 2006^51^	7.59	NR	
	Japan: Fujimoto, 2006^51^	18.57	NR	
	The Netherlands, multicentre: de Groot, 1996^50^	36.99	8.22	22.22

NR, not reported.

A subsample of studies reported both PPH and severe PPH (Table 3). The ratio of severe PPH to PPH should theoretically be similar regardless of how the third stage of labour was managed, unless a uterotonic has the characteristic of being more effective at preventing blood loss at lower or higher levels of blood loss. The ratio of severe PPH to PPH also varied from 10 to 33% for expectant management, from 6 to 41% for oxytocin, from 0 to 31% for misoprostol, and from 0 to 30% for ergometrine. In study sample arms with ≤200 women the range of severe PPH to PPH was 0–41%. In the larger study arms the range of severe PPH to PPH was slightly narrower: 4–33%.

### Association of the management of the third stage of labour with blood loss measured

#### Oxytocin versus expectant management

In all controlled studies of measured blood loss (Figure S1; Table 4), oxytocin significantly reduced PPH (OR 0.43, 95% CI 0.23–0.81; six studies, *n* = 6892), and reduced mean blood loss by 140 ml (95% CI from −228 to −52 ml; four studies, *n* = 2833) and was associated with substantially but not significantly lower rates of severe PPH (OR 0.61, 95% CI 0.29–1.29; six studies, *n* = 6892), compared with expectant management (no uterotonic prophylaxis). Significant heterogeneity (differences between studies) was observed in these results. Limiting the analyses to studies qualified as methodologically adequate eliminates the heterogeneity, as it reduces the analyses to one study (*n* = 1000). In adequate studies, oxytocin significantly reduced PPH (OR 0.45, 95% CI 0.34–0.60), and reduced the mean blood loss by 118 ml (95% CI from −165 to −71 ml). The association with severe PPH is marginally significant (OR 0.76, 95% CI 0.52–1.09). Similarly, oxytocin substantially but not significantly lowered severe PPH in the two studies conducted in developing countries, both of which were quasi-experimental (and thus did not qualify as methodologically adequate) (OR 0.42, 95% CI 0.04–4.81; two studies, *n* = 5203).^26^,^32^

**Table 4:** Effect of prophylactic regimen of third stage of labour on PPH, severe PPH and mean blood loss (in ml)

Outcome	Studies	*n*	Effect estimate OR/mean difference [95% CI]	*P*	Studies	*n*	Effect estimate OR/mean difference [95% CI]	*P*
				
	All studies				Adequate quality RCT subgroup			
**Oxytocin versus no uterotonic**								
PPH	6	6892	0.43 [0.23, 0.81]*	<0.001	1	1000	0.45 [0.34, 0.60]	<0.001
Severe PPH	6	6892	0.61 [0.29, 1.29]*	0.20	1	1000	0.76 [0.52, 1.09]	0.12
Mean blood loss	4	2833	−140.35 [−228.54, −52.16]*	0.001	1	1000	−118.00 [−165.23, −70.77]	<0.001
**Misoprostol versus no uterotonic**								
PPH	3	2687	0.73 [0.50, 1.08]*	0.12	2	2281	0.63 [0.41, 0.99]**	0.04
Severe PPH	6	4328	0.74 [0.52, 1.04]	0.09	5	3922	0.67 [0.51, 0.89]	0.005
Mean blood loss	3	2373	−38.75 [−64.81, −12.70]	0.004	3	2373	−38.75 [−64.81, −12.70]	0.004
**Ergometrine versus no uterotonic**								
PPH	2	1718	0.46 [0.11, 1.91]*	0.29				
Severe PPH	2	1718	0.32 [0.04, 2.43]**	0.27				
Mean blood loss	2	1718	−84.07 [−102.47, −65.67]	<0.001				
**Oxytocin versus misoprostol**								
PPH	5	20868	0.65 [0.60, 0.70]	<0.001	3	19139	0.65 [0.60, 0.70]	<0.001
Severe PPH	4	19789	0.71 [0.56, 0.91]	0.005	2	18941	0.70 [0.60, 0.83]	<0.001
Mean blood loss	3	2209	−8.36 [−18.32, 1.61]	0.10	1	200	−16.70 [−36.96, 3.56]	0.11
**Oxytocin versus ergometrine**								
PPH	3	1619	0.72 [0.34, 1.56]	0.41				
Severe PPH	1	1293	0.73 [0.20, 2.59]	0.63				
Mean blood loss	3	1619	−36.97 [−106.47, 32.53]*	0.30				
**Misoprostol versus ergometrine**								
PPH	3	2834	0.91 [0.67, 1.23]	0.53	1	1228	0.90 [0.63, 1.28]	0.56
Severe PPH	2	2634	0.30 [0.08, 1.15]	0.08	1	1228	0.47 [0.09, 2.60]	0.39
Mean blood loss	2	2634	−1.64 [−16.50, 13.22]	0.83	1	1228	−11.00 [−30.75, 8.75]	0.28

* Significant heterogeneity.

** Borderline significant heterogeneity (*P* = 0.06–0.10).

#### Misoprostol versus expectant management

Compared with no uterotonic prophylaxis, misoprostol was marginally associated with a substantial reduction in PPH (OR 0.73, 95% CI 0.50–1.08, three studies, *n* = 2687) and severe PPH (OR 0.74, 95% CI 0.52–1.04, six studies, *n* = 4328), and was significantly associated with a lower mean blood loss (−38.75 ml, 95% CI from −64.81 to −12.70 ml, three studies, *n* = 2833; Figure S2; Table 4). Significant heterogeneity was observed in the analysis of PPH. Limiting the analyses to studies qualified as methodologically adequate reduces the heterogeneity to marginally significant (*P*= 0.06), and confirms the effect of misoprostol on reducing PPH compared with expectant management (OR 0.63, 95% CI 0.41–0.99, two studies, *n* = 2281); both studies were conducted in rural areas of developing countries. In adequate studies (all in developing countries), misoprostol also significantly reduces severe PPH (OR 0.67, 95% CI 0.51–0.89, five studies, *n* = 3922) and mean blood loss (−39 ml, 95% CI from −65 to −13 ml, three studies, *n* = 2373).^24^,^28^,^42^,^43^,^52^

#### Ergometrine versus expectant management

Compared with no uterotonic, management with ergometrine was associated with a significant reduction in mean blood loss (−84 ml, 95% CI from −102 to −66 ml, two studies, *n* = 1718; Figure S3; Table 4). Whereas women receiving ergometrine had substantially lower PPH and severe PPH in all controlled studies (PPH, OR 0.46, 95% CI 0.11–1.91; severe PPH, OR 0.32, 95% CI 0.04–2.43, two studies, *n* = 1718), the differences were not statistically significant. None of the studies comparing ergometrine with expectant management was considered methodologically adequate, and none was conducted in developing countries.

#### Oxytocin versus misoprostol

Compared with misoprostol, oxytocin significantly reduced PPH (all controlled studies and adequate studies, OR 0.65, 95% CI 0.60–0.70, five studies, *n* = 20 868; Figure S4; Table 4) and severe PPH (all controlled studies, OR 0.71, 95% CI 0.56–0.91, four studies, *n* = 19 789; adequate studies, OR 0.70, 95% CI 0.60–0.83, two studies, *n* = 18 941). These odds ratios and 95% confidence limits of all studies and the adequate studies subgroup are identical, as the results are greatly influenced by the single WHO multicentre study.^45^ There was no considerable or significant difference between oxytocin and misoprostol in the two non-multicentre RCTs considered to be of adequate quality, which were conducted in much smaller tertiary care centres in developing countries (PPH, OR 0.83, 95% CI 0.51–1.37; severe PPH, OR 1.28, 95% CI 0.15–10.95, *n* = 1081) or in any of the studies solely conducted in developing countries (Figure S4). There was no difference in mean blood loss (all controlled studies, −8 ml, 95% CI from −18 to 2 ml, three studies, *n* = 2209; adequate studies, −17 ml, 95% CI from −37 to 4 ml, one study, *n* = 200). No statistical heterogeneity was observed in the comparisons. With the exception of one small study and the Ireland and Switzerland sites in the multicentre trial, these studies were conducted in developing country hospitals, none of which were in rural areas.

#### Oxytocin or misoprostol versus ergometrine

Oxytocin compared with ergometrine was associated with substantially lower PPH (oxytocin, OR 0.72, 95% CI 0.34–1.56, three studies, *n* = 1619) and severe PPH (oxytocin, OR 0.73, 95% CI 0.20–2.59, one study, *n* = 1293), although neither difference was statistically significant (Figure S5; Table 4). There was little difference in mean blood loss in women receiving oxytocin compared with ergometrine (−37 ml, 95% CI from −106 to 33 ml, three studies, *n* = 1619). In the single study in India (which was not of adequate quality), the results comparing oxytocin with ergometrine were almost identical to all studies comparing oxytocin with ergometrine (PPH, OR 0.71, 95% CI 0.35–1.43; severe PPH, OR 0.73, 95% CI 0.20–2.59; mean blood loss −5 ml, 95% CI from −20 to 10 ml, *n* = 1293).

Women who received misoprostol had similar PPH rates and mean blood loss to those receiving ergometrine (PPH, OR 0.91, 95% CI 0.67–1.23, three studies, *n* = 2834; mean blood loss −2 ml 95% CI from −17 to 13 ml, two studies, *n* = 2634; Figure S6; Table 4). However, women receiving misoprostol had substantially and marginally significantly lower rates of severe PPH than those receiving ergometrine (OR 0.30, 95% CI 0.08–1.15, *P*= 0.08, two studies, *n* = 2634). Only one study on rural Gambian home deliveries, comparing misoprostol with ergometrine, was considered to be adequate: there was no substantial difference in PPH or mean blood loss, but misoprostol was associated with a large yet not statistically significantly lower rate of severe PPH (OR 0.47, 95% CI 0.09–2.60, *n* = 1228).^53^ All studies comparing misoprostol with ergometrine were conducted in developing countries.

## Discussion

The WHO recommends oxytocin as the uterotonic of choice for PPH prevention, and that oxytocin or misoprostol be offered by a health worker trained in its use in the absence of oxytocin and other components of AMTSL, e.g. provision of a uterotonic, uterine massage and controlled cord traction.^8^ These recommendations are currently based upon a body of studies that do not distinguish between visual and measured blood loss, and are influenced by the sample for which blood loss was visually assessed. Similarly, analyses upon which policy recommendations are based do not separate studies for other factors that influence bleeding. The American College of Obstetricians and Gynecologists has suggested functional definitions of severe blood loss, including a 10% decline from ante- to post-partum haematocrit, or the need for red blood cell transfusion;^55^ however, too few studies measuring blood loss exist to support such functional definitions.

By reviewing only articles of measured postpartum blood loss, this article provides comparisons unbiased by the proportion of studies using visual compared with measured blood loss. Most of the presented analyses show similar effects to those published in meta-analyses that pool visually estimated and measured blood loss; however, our analyses clarify some important discrepancies.^17^ Comparing oxytocin with no uterotonic, our analyses of all studies show a slightly stronger and still significant effect for PPH and mean blood loss (PPH, OR 0.43, 95% CI 0.23–0.81 versus Cochrane OR 0.50, 95% CI 0.43–0.59; mean blood loss of −140 ml, 95% CI from −229 to −52 ml versus Cochrane blood loss of −102 ml, 95% CI from −135 to −69 ml), with the same effect on severe PPH (OR 0.61, 95% CI 0.29−1.29 versus Cochrane 95% CI 0.44–0.87). As a smaller subgroup, our analyses of severe PPH do not reach statistical significance.^17^ Our analyses of studies of adequate quality compared with the Cochrane subgroup of RCTs demonstrate a significant and much stronger reduction of PPH with oxytocin compared with no uterotonic (OR 0.45, 95% CI 0.34–0.60 versus Cochrane OR 0.61, 95% CI 0.51–0.72), and a reduction in mean blood loss (−118 ml, 95% CI from −165 to −71 ml versus Cochrane mean blood loss of −109 ml, 95% CI from −152 to −66 ml), whereas the effect on severe PPH was similar, and was still marginally significant (OR 0.76, 95% CI 0.52−1.09 versus Cochrane OR 0.72, 95% CI 0.49–1.05).

The Cochrane comparisons of misoprostol with no or other uterotonics are less methodologically similar to our analyses.^8^ The Cochrane review of prostaglandins for PPH prevention does not provide estimates summarising the overall effect comparing misoprostol with no uterotonic; however, the estimate we calculate from the data they present for this comparison, excluding the Gambian study^53^ (as the comparison group received oral ergometrine) and the Turkish study^36^ (which compared a combination of oxytocin and misoprostol with no uterotonic), was Cochrane OR 0.75 (95% CI 0.49–1.14) for severe PPH, very similar to our results from all studies (OR 0.74, 95% CI 0.52–1.04), although our adequate-quality studies showed a stronger and highly significant effect (OR 0.67, 95% CI 0.51–0.89). The Cochrane review found that compared with sublingual misoprostol, any injectable uterotonic had a similar yet marginally significant effect on PPH (Cochrane OR 0.93, 95% CI 0.79–1.11), and was inferior to sublingual misoprostol for severe PPH (Cochrane OR 1.85, 95% CI 0.79–4.35). In contrast, the Cochrane review found any injectable uterotonic to be superior to oral misoprostol for severe PPH (Cochrane OR 0.76, 95% CI 0.66–0.86).^8^

The effects of uterotonics on severe PPH are particularly important, as maternal death as a result of PPH usually occurs when blood loss is >1000 ml.^19^ Distinct from existing reviews, we found that prophylactic oxytocin significantly reduces PPH, but is only marginally associated with lower severe PPH compared with expectant management. This might be attributable to insufficient statistical power, as severe PPH is a relatively rare condition. In addition, a small portion of PPH and severe PPH would not be responsive to uterotonics (for example, if caused by trauma), thereby minimising the incidence of potentially responsive severe bleeding. However, compared with no uterotonic, misoprostol significantly lowered severe PPH in adequate-quality studies with a much smaller total sample size than that of all studies evaluating the effects of oxytocin on severe PPH. The data from adequate-quality studies or developing country data are too scant to draw conclusions about the effects of oxytocin compared with no uterotonic, or misoprostol, in these contexts.

Prophylactic misoprostol significantly reduces PPH and severe PPH, compared with expectant management, only when analyses are limited to adequate-quality studies, or in studies solely conducted in developing countries. In the WHO multicentre study comparing oxytocin with misoprostol in hospital settings, oxytocin reduces PPH and severe PPH significantly more than misoprostol, but does not differentially affect maternal death.^45^ Four studies of misoprostol have been conducted in rural, developing country settings: two compared with ergometrine and two with no uterotonic. There is only one quasi-experimental study of oxytocin in a rural developing country setting. No studies compare oxytocin with misoprostol in home birth or primary care centre settings, or in rural areas of developing countries, where misoprostol being simpler, and therefore more feasible to administer and study, may be relatively more effective because of greater coverage.

Distinct from other reviews, this review of measured blood loss, complementing meta-analyses with broader epidemiologic data, and providing sufficient stratification of information, demonstrates that women experience a large range of postpartum blood loss, even when bleeding was carefully measured. The median of reported average blood loss in women receiving any prophylactic uterotonics was similar, and was approximately 40% lower than that of women not receiving prophylactic uterotonics. However, the range of average blood loss, PPH and severe PPH was large, and fairly consistent, across women receiving and not receiving prophylactic uterotonics. The difference between the lowest and highest mean blood loss, incidence of PPH and severe PPH, and the ratio of severe PPH to PPH within each regimen for managing the third stage of labour is greater than the discrepancy in these ranges across the regimens. Variation in blood loss was only slightly larger in study arms with ≤200 women compared with larger study arms.

Women’s characteristics, obstetric practices and other factors associated with setting could account for some of the blood loss variation, and for the differences in the relative effectiveness of uterotonics on blood loss and haemorrhage.^10^ Eligible studies generally excluded high-risk or complicated pregnancies. Labour augmentation and/or induction were permitted in about half of the reviewed studies with 2–47% (median 27%) of women having augmentation or induction. In women otherwise managed without uterotonic prophylaxis, study arms that permitted augmentation or induction had lower levels of blood loss than those without. Measuring blood loss is more difficult than visual estimation, and thus has been implemented less frequently. Although blood loss measurement could in theory influence observed blood loss, few studies used the drape, and both the bedpan/linens and drape methods are direct measurements that are found to be quite accurate and similar.^56^,^57^ Exclusion of studies where the incidence of PPH was extremely high, or limiting analyses to studies measuring blood loss for 1 hour, only slightly modified the incidence of severe PPH for all regimens.

## Conclusions

A better understanding of postpartum blood loss could improve our strategies to prevent and manage PPH, particularly in the rural developing country settings where most maternal deaths occur, yet where few adequate-quality studies have taken place. Our results of measured blood loss indicate that although oxytocin is superior to misoprostol in hospitals, misoprostol substantially lowers PPH and severe PPH in developing countries. The relative merits of oxytocin and misoprostol continue to require sound assessment in rural areas of developing countries, where most PPH deaths occur.

## Supporting information

The following supplementary materials are available for this article:

**Figure S1.** Oxytocin versus expectant management.

**Figure S2.** Misoprostol versus expectant management.

**Figure S3.** Ergometrine versus expectant management.

**Figure S4.** Oxytocin versus misoprostol.

**Figure S5.** Oxytocin versus ergometrine.

**Figure S6.** Misoprostol versus ergometrine.
