# Supplementary material for: What measured blood loss tells us about postpartum bleeding: a systematic review
Source: BJOG. 2010 Jun;117(7):788–800. doi: 10.1111/j.1471-0528.2010.02567.x (PMC2878601; doi:10.1111/j.1471-0528.2010.02567.x)
Supplement: Supplementary file 1 [file bjo0117-0788-SD1.doc]

Figure S1: Oxytocin v. Expectant Management

Outcome: 3.1 PPH

Developing country subset: OR = 0.30, 95% CI 0.06, 1.51, p=0.14

Outcome: 3.2 Severe PPH

Developing country subset: OR = 0.42, 95% CI 0.04, 4.81, p=0.48

Outcome: 3.3 Mean Blood Loss.

Developing country subset: Mean blood loss-221.20, , 95% CI -246.69, -195.71, p≤0.001

(R) signifies a rural setting
