# Supplementary material for: What measured blood loss tells us about postpartum bleeding: a systematic review
Source: BJOG. 2010 Jun;117(7):788–800. doi: 10.1111/j.1471-0528.2010.02567.x (PMC2878601; doi:10.1111/j.1471-0528.2010.02567.x)
Supplement: Supplementary file 2 [file bjo0117-0788-SD2.doc]

Figure S2: Misoprostol v Expectant Management

Outcome 4.1 PPH.

Developing country subset: OR = 0.63, 95% C.I. 0.41, 0.99, p=0.04

Outcome: 4.2 Severe PPH

Developing country subset: OR = 0.67, 95% C.I 0.51, 0.89, p=0.005

Outcome: 4.3 Mean Blood Loss.

All are developing countries

(R) signifies a rural setting
