# Supplementary material for: What measured blood loss tells us about postpartum bleeding: a systematic review
Source: BJOG. 2010 Jun;117(7):788–800. doi: 10.1111/j.1471-0528.2010.02567.x (PMC2878601; doi:10.1111/j.1471-0528.2010.02567.x)
Supplement: Supplementary file 3 [file bjo0117-0788-SD3.doc]

Figure S3: Ergometrine v Expectant Management

Outcome 5.1 PPH.

None are Developing Countries

Outcome: 5.2 Severe PPH

None are Developing Countries

Outcome: 5.3 Mean Blood Loss.

None are Developing Countries
