# Supplementary material for: What measured blood loss tells us about postpartum bleeding: a systematic review
Source: BJOG. 2010 Jun;117(7):788–800. doi: 10.1111/j.1471-0528.2010.02567.x (PMC2878601; doi:10.1111/j.1471-0528.2010.02567.x)
Supplement: Supplementary file 4 [file bjo0117-0788-SD4.doc]

Figure S4: Oxytocin v Misoprostol

Outcome 6.1 PPH.

Developing country subset: OR = 0.82, 95% CI 0.55, 1.24, p=0.35

Adequate studies excluding Multicentre Gulmezoglu 2001: OR=0.83, 95% CI 0.51 - 1.37, p=0.47

Outcome: 6.2 Severe PPH

Developing country subset: OR = 1.28, 95% CI 0.15, 10.95, p=0.82

Adequate studies excluding Multicentre Gulmezoglu 2001: OR=1.28, 95% CI 0.15 to 10.95, p=0.82

Outcome: 6.3 Mean Blood Loss.

All are developing countries

Adequate studies excluding Multicentre Gulmezoglu 2001: Mean blood loss-11.82, 95% C.I.-23.33, -0.31, p=0.04
