# Supplementary material for: What measured blood loss tells us about postpartum bleeding: a systematic review
Source: BJOG. 2010 Jun;117(7):788–800. doi: 10.1111/j.1471-0528.2010.02567.x (PMC2878601; doi:10.1111/j.1471-0528.2010.02567.x)
Supplement: Supplementary file 5 [file bjo0117-0788-SD5.doc]

Figure S5: Oxytocin v Ergometrine

Outcome 7.1 PPH.

Developing country subset: OR 0.71, 95% C.I. [0.35, 1.43, p=0.33

Outcome: 7.2 Severe PPH

All are developing countries

Outcome: 7.3 Mean Blood Loss.

Developing country subset: Mean blood loss -5.00, 95% C.I. -19.61, 9.61, p=0.50
