# Supplementary material for: What measured blood loss tells us about postpartum bleeding: a systematic review
Source: BJOG. 2010 Jun;117(7):788–800. doi: 10.1111/j.1471-0528.2010.02567.x (PMC2878601; doi:10.1111/j.1471-0528.2010.02567.x)
Supplement: Supplementary file 6 [file bjo0117-0788-SD6.doc]

Figure S6: Misoprostol v Ergometrine

Outcome 8.1 PPH.

All are developing countries

Outcome: 8.2 Severe PPH

All are developing countries

Outcome: 8.3 Mean Blood Loss.

All are developing countries

(R) signifies a rural setting
